# Editorial: Advances on the Biological Mechanisms Involved in Adventitious Root Formation: From Signaling to Morphogenesis

**DOI:** 10.3389/fpls.2022.867651

**Published:** 2022-02-28

**Authors:** Hélia Cardoso, Augusto Peixe, Catherine Bellini, Sara Porfírio, Uwe Druege

**Affiliations:** ^1^MED – Mediterranean Institute for Agriculture, Environment and Development, Institute for Advanced Studies and Research, University of Évora, Évora, Portugal; ^2^MED – Mediterranean Institute for Agriculture, Environment and Development, Department of Plant Science, School of Science and Technology, University of Évora, Évora, Portugal; ^3^Umeå Plant Sciences Centre, Department of Plant Physiology, Umeå University, Umeå, Sweden; ^4^INRAE, AgroParisTech, Institut Jean-Pierre Bourgin (IJPB), Université Paris-Saclay, Versailles, France; ^5^Complex Carbohydrate Research Center, University of Georgia Athens, Athens, GA, United States; ^6^Erfurt Research Centre for Horticultural Crops, University of Applied Sciences Erfurt, Erfurt, Germany

**Keywords:** vegetative propagation, cell reprogramming, hormone crosstalk, reactive oxygen species (ROS), transcription factors, rooting recalcitrance

The neoformation of adventitious roots in cuttings is the key in the success of plant vegetative propagation systems. Two main steps are recognized in adventitious root (AR) formation. The first is known as the induction phase, in which high auxin levels are required for reprogramming of competent cells toward root development, which is further linked to other biochemical changes (Ahkami et al., 2013; Druege et al., 2019). This stage is followed by the formation phase, when cell division, differentiation and growth occur, leading to AR primordium development and, finally, to AR emergence/expression (Da Costa et al., Druege et al., 2019). Based on fossil records and phylogenetic data, Mhimdi and Pérez-Pérez proposed AR formation as the default state of root development in plants, highlighting that root initiation from non-root tissues might be considered as an ancestral trait, as ARs are formed by default in lycophytes and ferns. According to the same authors, in many angiosperms, AR formation seems to be repressed through mechanisms that maintain competent cells in a quiescent state. Activation of competent cells is triggered by environmental conditions to which the cuttings or intact plants are exposed, like exogenous auxin treatment, wounding, waterlogging, or light exposure and mineral nutrition determining the activation of a complex network of signaling, metabolic, and transport pathways (Bannoud and Bellini, Mhimdi and Pérez-Pérez, Yang et al., 2019). All these network pathways are under the control of distinct genes, being at the end responsible for efficient cell reprogramming, acquisition of a new evolutionary pattern and *de novo* organ differentiation and development.

The articles of Bannoud and Bellini, Mhimdi and Pérez-Pérez and Abarca, integrate recent findings on the molecular and genetic control of AR formation in several plant species and provide concepts about the molecular pathways, while Li, presented a list of genes involved in AR formation, as well as conceptional models for the interactions of the most important molecular players. All those authors considered the important role of initial auxin accumulation and its canalization toward the AR source cells during AR induction, referring to *ASA* and *YUCCA* genes, that encode anthranilate synthases and flavin monoxygenases, as the most important on the control of auxin biosynthesis, the *Gretchen Hagen 3* (*GH3*) genes, on the control of auxin or jasmonic acid conjugation, and the *PIN, AUX/LAX*, and *ABCB* genes, on the control of auxin transport.

Important molecular factors that determine auxin signaling are specific *Aux/IAA* and *ARF* genes that encode auxin signaling repressors and their target auxin response factors respectively. Downstream, genes of transcription factors (TFs) from the *GRAS, SHORTROOT* (*SHR*), *SCARECROW* (*SCR*), *WUSCHEL RELATED HOMEOBOX* (*WOX*), *LATERAL ORGAN BOUNDARIES-DOMAIN* (*LOB*) and *APETALA2/ETHYLENE RESPONSIVE ELEMENT BINDING* (*AP2*/*ERF*) gene families, control the auxin-dependent establishment of the AR founder cell and the new root apical meristem, the maintenance of the quiescent center (QC) and subsequent establishment and differentiation of the AR primordium (Li, Bannoud and Bellini, Mhimdi and Pérez-Pérez, Abarca). A coordinated change of expression of genes controlling the modulation of the cell wall, such as *EXTENSIN* and CYS *ENDOPEPTIDASE* genes, may probably also be involved (Li). Even though these reviews present different perspectives, they stay in line to each other in pinpointing to the core function of plant hormone crosstalk on regulating AR formation, within a process involving a hierarchy of transcription factors, that is linked to epigenetic modulation events, with reactive oxygen species (ROS) also being involved as messenger molecules.

The involvement of enzymes from ROS-scavenging mechanisms, and phenolic compounds working as a non-enzymatic antioxidant system, have been investigated across a diversity of plant species during the AR formation process. The activity of ascorbate peroxidase (APX), superoxide dismutase (SOD), and catalase (CAT) have been associated with improved rooting ability (Macedo et al., 2012; Niu et al., 2017; Velada et al., 2018). Vilasboa et al. highlight the capacity to efficiently modulate redox conditions though high activity of SOD and soluble GPRX, as the main factor defining AR ability in *Eucalyptus*. The role of phenolic compounds acting as synergistic or antagonistic in AR formation has long been known, and its role as a species-dependent parameter was also highlighted by Vilasboa et al.

Within the different ROS molecules, the focus has been given to H_2_O_2_, acting as second messenger in phytohormone signaling. The role of H_2_O_2_ in promoting AR formation has been demonstrated in different plant species including poplar (Macedo et al., 2009, Bannoud and Bellini). The importance of ROS on AR formation was also explored by studying the involvement of the alternative oxidase (AOX), a key enzyme in the alternative respiratory pathway, using semi-hardwood cuttings (Macedo et al., 2009, 2012) and explants from *in vitro* cultured plants (Velada et al., 2018). The activity of peroxidases, a group of enzymes that catalyze the oxidation of a substrate by H_2_O_2_, some of them responsible by canalization of IAA, was also correlated with rooting process and the monitorization of peroxidases activity as markers for rooting ability was highlighted (Porfírio et al., 2016, Bannoud and Bellini).

The loss of rooting capacity with plant aging (maturation-induced rooting recalcitrance), a major problem in breeding and propagation of many tree species, has also been investigated. Regarding the relevant literature, Abarca suggested the epigenetic control of miR160/167-modulated auxin signaling, the *TARGET OF RAPAMICINE (TOR)* and possibly *RETINOBLASTOMA-RELATED (RBR)* dependent metabolic and auxin control of the cell cycle, and the non-cell autonomous regulation of *WOX11/12* expression by CLAVATA3/EMBRYO SURROUNDING REGION-RELATED (CLE) peptides, as three molecular checkpoints for the age-related decline in AR induction. In their original article, Pizarro and Díaz-Sala compared hypocotyl cuttings excised from *Pinus radiata* seedlings with different ages to identify genes that control the cell wall-plasma membrane-cytoskeleton continuum in the basal stem, in relation to rooting competence. The *RECEPTOR-LIKE KINASE* (*RLK*) and *MECHANOSENSITIVE CHANNEL* (*MSC*) genes, as well as genes encoding downstream regulators of the small GTP-binding protein family, were associated with rooting competence, probably *via* acting on the control of cell wall sensing and membrane disturbances during the essential cellular reorganization (Pizarro and Díaz-Sala).

Despite all the research carried out and the information achieved in the last years, the mechanisms underlying maturation-induced rooting recalcitrance and environmentally mediated rooting, as well as several other aspects of the AR formation process, are still far from being fully understood (Druege et al., 2019, Bannoud and Bellini, Abarca).

## Author Contributions

HC, AP, and UD: writing of the original draft. HC, AP, UD, SP, and CB: review and edition of the final version. All authors contributed to the article and approved the submitted version.

## Funding

The work of HC and AP was funded by National Funds through FCT—Foundation for Science and Technology under the Project UIDB/05183/2020 and by FEDER and National Funds through the ALENTEJO 2020 - Project OLEAVALOR (ALT20-03-0145-FEDER-000014). The work of UD was funded by the German Federal Ministry of Food and Agriculture (BMEL) based on a decision of the Parliament of the Federal Republic of Germany, granted by the Federal Office for Agriculture and Food (BLE; grant number 2818HSE02).

## Conflict of Interest

The authors declare that the research was conducted in the absence of any commercial or financial relationships that could be construed as a potential conflict of interest.

## Publisher's Note

All claims expressed in this article are solely those of the authors and do not necessarily represent those of their affiliated organizations, or those of the publisher, the editors and the reviewers. Any product that may be evaluated in this article, or claim that may be made by its manufacturer, is not guaranteed or endorsed by the publisher.
